# 

**Published:** 1898-07-23

**Authors:** 


					THE HOSPITAL. "The standard of highest purity."?Lancet. July 23rd, 1898. A
CADBURY'S mA ? tee*' CADBURY'S
cogoa Jrll m cocoa
ABSOLUTELY PURE. W?t ' Z" ~ NO DRUGS OR ALKALIES USED.
W/CH HflSPl TA L
^?^CiLASCnw Wr<;TF?*J Infirmary
No. 617. Vol. XXIV. Saturday,
July 23rd, 1898.
^Subscription^Terms,] pRICE TWOPENCE.

				

